# EMT Factors and Metabolic Pathways in Cancer

**DOI:** 10.3389/fonc.2020.00499

**Published:** 2020-04-07

**Authors:** Ilias Georgakopoulos-Soares, Dionysios V. Chartoumpekis, Venetsana Kyriazopoulou, Apostolos Zaravinos

**Affiliations:** ^1^Department of Bioengineering and Therapeutic Sciences, University of California, San Francisco, San Francisco, CA, United States; ^2^Institute for Human Genetics, University of California, San Francisco, San Francisco, CA, United States; ^3^Service of Endocrinology, Diabetology and Metabolism, Lausanne University Hospital and University of Lausanne, Lausanne, Switzerland; ^4^Division of Endocrinology, Department of Internal Medicine, School of Medicine, University of Patras, Patras, Greece; ^5^College of Medicine, Member of QU Health, Qatar University, Doha, Qatar; ^6^Department of Life Sciences European University Cyprus, Nicosia, Cyprus

**Keywords:** EMT, metabolic pathways, transcription factors, non-coding RNAs, cancer metabolism

## Abstract

The epithelial-mesenchymal transition (EMT) represents a biological program during which epithelial cells lose their cell identity and acquire a mesenchymal phenotype. EMT is normally observed during organismal development, wound healing and tissue fibrosis. However, this process can be hijacked by cancer cells and is often associated with resistance to apoptosis, acquisition of tissue invasiveness, cancer stem cell characteristics, and cancer treatment resistance. It is becoming evident that EMT is a complex, multifactorial spectrum, often involving episodic, transient or partial events. Multiple factors have been causally implicated in EMT including transcription factors (e.g., SNAIL, TWIST, ZEB), epigenetic modifications, microRNAs (e.g., miR-200 family) and more recently, long non-coding RNAs. However, the relevance of metabolic pathways in EMT is only recently being recognized. Importantly, alterations in key metabolic pathways affect cancer development and progression. In this review, we report the roles of key EMT factors and describe their interactions and interconnectedness. We introduce metabolic pathways that are involved in EMT, including glycolysis, the TCA cycle, lipid and amino acid metabolism, and characterize the relationship between EMT factors and cancer metabolism. Finally, we present therapeutic opportunities involving EMT, with particular focus on cancer metabolic pathways.

## The Epithelial to Mesenchymal Transition (EMT) Process

In recent years, it has been progressively realized that cell identity is highly dynamic, as most notably demonstrated by Yamanaka et al., by reprogramming fully differentiated fibroblasts into induced pluripotent stem cells with the induction of four transcription factors (TFs) (1). Stem cells can renew themselves while maintaining their multipotency or can differentiate to a less potent cell type. During development and embryogenesis, a cascade of epigenetic and transcriptional programs is employed to ensure the differentiation of multipotent progenitor cells. Epithelial to mesenchymal transition (EMT) represents a cell biological program, during which epithelial cells progressively miss their cell identity and morphology and increasingly acquire mesenchymal characteristics (2, 3). The converse route is recognized as mesenchymal to epithelial transition (MET). EMT and MET are both mediated through a cascade of transcriptional and epigenetic changes and are physiologically observed during organismal development, tissue healing, homeostasis, as well as during fibrosis. However, the same processes can be hijacked by tumor cells during cancer development (4). Indeed, several analogies have been drawn between organismal development and tumorigenesis (5).

Epithelial cells exhibit cell-cell junctions, apico-basal polarity and limited migratory potential, and they can be identified using a multitude of cell surface markers, most notably E-cadherin, but also cytokeratins, occluding, and claudins (6, 7). In contrast, mesenchymal cells are characterized by front rear polarity and a migratory phenotype. In addition, during cancer development they display resistance to apoptosis, stem cell characteristics and tissue invasiveness (2, 8, 9). Mesenchymal cells can be identified by N-cadherin, Fibronectin and Vimentin cell surface markers (6). The EMT/MET model proposes that the migratory phenotype of cancer cells is acquired during EMT, enabling the invasion of other tissues, while MET potentiates the settlement of cancer cells at the new site (6).

Current work has demonstrated that EMT is not a binary state procedure, during which cells can either have a mesenchymal or an epithelial identity. Instead, EMT is a dynamic spectrum and reversible process and cells can be found at any locale on that spectrum, often sharing certain epithelial and mesenchymal characteristics (10–15). Most importantly, cells considered to be in a hybrid epithelial/mesenchymal state are more apoptosis-resistant and have higher tumor-initiating potential (15–17) Technological advances in CRISPR-Cas9 genome editing and decreasing costs in single cell sequencing have potentiated important breakthroughs in EMT. Firstly, single cell sequencing has revealed the extensive variability in gene expression and cell identity during EMT, both in development (18) and cancer (14, 19). Secondly, recent studies have demonstrated partial and transient EMT events with cells found across the continuum along the EMT spectrum (19–22). Thirdly, the variability and complexity in the expression patterns of multiple EMT and MET factors across disparate cell types and conditions is being appreciated and novel players in the EMT process are being discovered (14, 18, 19, 21, 22). CRISPR-Cas9 screens coupled with single cell RNA sequencing have identified novel EMT-associated factors and have provided evidence that specific signaling pathways control the EMT via discrete, regulatory checkpoints (21).

Cancer cells are known to adapt their metabolism to meet their high needs for energy and synthesis of biomolecules including proteins, lipids and nucleic acids (23, 24). Tumor cells are usually characterized by the Warburg effect, that is, the production of ATP mostly from glycolysis and not oxidative phosphorylation, even in conditions with high availability of oxygen (25). However, a multitude of key metabolic pathways are involved in the metabolic adaptations of cancer cells, with accumulating evidence for the importance of these pathways in EMT. Most notable among them are glycolysis, the TCA cycle, lipid and amino acid metabolism, which directly contribute to EMT, cancer cell survival, cancer invasiveness and metastasis. Although the regulation of these metabolic pathways was considered to be largely known, it seems that recent advances in our capacity to measure specific metabolites at the cell level and especially in the cancer state have shed new light on their modulation and intertwining with EMT transcriptional regulation (26, 27). As epithelial cancer cells acquire mesenchymal features during the EMT process, their metastatic potential increases. As a result, they should be able to penetrate the extracellular matrix, enter the blood stream and finally grow in a different tissue. All these steps require a continuous supply of nutrients to the cells that is provided through the blood stream and by metabolic reprogramming of the cells (28). More evidence is accumulating that this metabolic reprogramming is a highly regulated process by transcription factors that are known to be involved in EMT (29).

The requirement of EMT for metastasis may rely on the cancer type and there is ongoing contention regarding its role in metastasis, which may also be context-dependent and transient. There is substantial evidence supporting the notion that EMT is a driver during cancer metastasis in certain cancer types (30–37). Additionally, the induction of MET and the down-regulation of EMT TFs at the site of metastasis, supports the colonization of the metastatic cells (38–40). Metastasis accounts for an estimated 90% of cancer-associated deaths (41), reinforcing the importance of intervention at EMT. Various signaling molecules can activate the EMT process, including epidermal growth factor (EGF), fibroblast growth factor (FGF), hepatocyte growth factor (HGF), transforming growth factor β (TGFβ), β-catenin–dependent canonical and β-catenin–independent non-canonical WNT signaling, bone morphogenetic protein (BMP), Sonic Hedgehog (SHH) and the Notch signaling pathway, among others (12, 42–46). EMT transcription factors, epigenetic alterations, microRNAs, post-translational modifications, and metabolic reprogramming orchestrate the transition. In this review, we delve into each of them from a molecular and cellular viewpoint and summarize recent advances and changes in our understanding.

## EMT- Transcription Factors (TFs) and Signaling That Regulates the EMT Process

EMT-TFs represent master TFs that coordinate the EMT process. The most widely studied TFs among them are TWIST1, TWIST2, SNAIL1, SNAIL2, ZEB1, and ZEB2 (12), all of which directly inhibit the expression of E-cadherin and promote the transition to a mesenchymal state. The consequence of their expression is the suppression of the epithelial phenotype and the associated loss of epithelial cell surface biomarkers. A common feature among EMT-TFs is their physiological roles in embryogenesis and organismal development, as well as their reappearance in cancer cells during cancer development and progression. The expression of EMT-TFs can be overlapping and they can form networks, yet their functions are usually distinct. They are activated through signaling cascades and promote the transcriptional program switching. EMT-TFs have clinical relevance in metastasis and their expression correlates with poor clinical outcomes in cancer (6, 47, 48).

### The SNAIL1 and SNAIL2 Master TFs in EMT

The SNAIL sub-family within the larger SNAG domain family of Zinc finger TFs in humans is composed of three members, namely SNAIL1, SNAIL2 (also known as SLUG), and SNAIL3, which act as transcriptional repressors (49). The number of SNAIL members varies by species and they are usually associated with mesoderm development and differentiation (50) and wound healing (51). SNAIL1 and SNAIL2 have important and widely studied roles in the EMT process, whereas SNAIL3 is a paralogue of SNAIL1 and SNAIL2 with distinct and divergent functions (52). SNAIL1 and SNAIL2 downregulate the expression of a number of target genes in relation to EMT, most notably E-cadherin, but also claudins, occludin, PALS1 and PATJ (53, 54). Both SNAIL1 and SNAIL2 bind directly to the E-cadherin promoter at E-box sequences to inhibit its expression (55). SNAIL1 interacts with chromatin remodeling factors to exert its repressor activity at the E-cadherin promoter (56). It also alters the polarity of epithelial cells by inhibiting the expression of Crumbs3, which is essential for epithelial morphogenesis (57). In addition to mediating the EMT process, SNAIL members promote cell survival, block the cell cycle and inhibit the apoptotic process, with roles in the induction of a metastatic phenotype and the acquisition of cancer stem cell features (8, 58). In support to that, circulating tumor cells from hepatocellular carcinomas express roughly 10 times more SNAIL1 mRNA (59), while transient SNAIL2 and SOX9 induction increases the metastatic ability of mammary gland cells (60).

**Figure 1:**
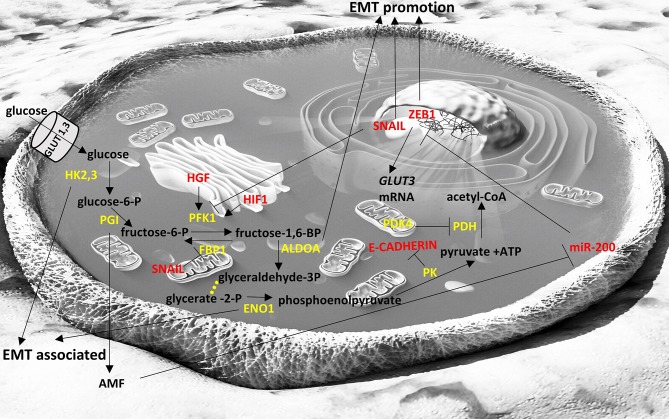
Interplay of glycolysis pathway with EMT factors. Enzymes are depicted in yellow font, EMT-related factors are depicted in red font. → denotes induction; ⊣ denotes inhibition. Yellow dots indicate intermediate reactions that are not depicted.

A plethora of general and cell-type specific signals can activate SNAIL TFs. TGFβ1 induces SNAIL1 expression in a number of cell types including hepatocytes, palate, epithelial and mesothelial cells (61, 62). TGFβ2 induces SNAIL1 expression during hair follicle morphogenesis (63) and SNAIL2 expression during heart development (64). BMP4 induces SNAIL2 expression during neural crest development (65). Snail genes are up-regulated in multiple cancer types and they are associated with poor prognosis, including breast and ovarian cancers for SNAIL1 (66, 67) and colorectal cancers for SNAIL2 (68). In pancreatic and thyroid cancers and their metastases Snail genes are upregulated (69, 70). Furthermore, SNAIL TFs promote cancer recurrence (71) and resistance to cancer treatments (72). In turn, SNAIL1 can induce changes in the metabolism of glucose and can control the dependence of cancer cells to glycolysis relative to the pentose phosphate pathway (73), indicating the link between EMT factors, metabolism and cancer cell survival (Figure 1).

Phosphorylation of SNAIL proteins is crucial for their localization and their functionality in the cell (74). Glycogen Synthase Kinase-3 (GSK3) is a kinase that phosphorylates SNAIL1 resulting in its nuclear export and degradation (75). GSK3 can inhibit the EMT process by targeting SNAIL1 across multiple cell types (76). GSK3's activity contrasts that of p21-activated kinase (PAK1) which phosphorylates SNAIL1 resulting in its nuclear localization and the activation of EMT (77). Indeed, SNAIL1 expression and activation induces fibrosis in kidney and EMT (78). Importantly, de-phosphorylation of SNAIL proteins by Small C-terminal domain phosphatase (SCP) can also affect their localization and activity (79). Stabilization of SNAIL1 by nuclear factor kappa B (NF-kB) is mediated through prevention of its phosphorylation by GSK3 (80). Therefore, multiple post-translational regulators of SNAIL proteins control their functions and are putative targets for intervention.

### Basic Helix Loop Helix EMT-TFs TWIST1 and TWIST2

TWIST1 and TWIST2 belong to the family of basic helix loop helix (bHLH) TFs. TWIST proteins are structurally similar and bind to E-box DNA response elements to repress or activate transcription (81). They have important physiological roles during embryonic morphogenesis, wound healing and tissue fibrosis (82, 83). In contrast, they are not expressed, or are expressed at extremely low levels, in most cell types after embryogenesis with the exception of certain precursor cell types (84), suggesting that they could be an attractive target for therapeutics or cancer biomarker development.

Expression of TWIST TFs can induce the EMT process and they are upregulated during cancer development (85, 86) and progression to metastasis (87). TWIST TFs are also associated with worse patient prognosis (87). Increasing the expression of TWIST1 is directly associated with tumor invasion and metastasis and mediates the loss of E-cadherin, a key epithelial marker. It also increases the expression of the mesenchymal markers Fibronectin, N-cadherin and Vimentin, leading to the reduction of cell adhesion and the promotion of cellular motility (87, 88). TWIST proteins also promote a cancer stem cell phenotype (89). TWIST activity can be modulated via post-translational modifications, such as phosphorylation. TWIST1 phosphorylation by MAP kinase stabilizes the protein and promotes breast cancer cell invasiveness and EMT (90). Similarly, AKT-mediated phosphorylation of TWIST results in increased invasiveness (91). In contrast, phosphorylation by IKKβ results in the degradation of TWIST (92).

The role of TWIST in metabolism has been mainly described in adipose tissue and has been associated with increased inflammation and insulin resistance (93). Its role in cancer metabolism has not been elucidated but it seems to be activated by asparagine and promote EMT (**Figure 4**) (94).

Another member of the bHLH EMT-TF group is Transcription Factor 3 (TCF3 or E2A) which produces two splice variants, E47 and E12 (95). TCF3 can induce the EMT process by inhibiting the expression of E-cadherin (96). Finally, E2.2 (also known as TCF4) is not a master EMT-TF, but it can induce a full EMT and represses E-cadherin expression indirectly, through complex, functional and hierarchical interactions with EMT factors (97, 98). Indeed, E2.2 is upregulated in cells overexpressing SNAIL1, SNAIL2, or E47 and after inhibition of E2.2 expression, the EMT is maintained, when driven by SNAIL1 and E47 (97).

### Zinc-Finger E-Box Binding (ZEB) 1/2

The ZEB family in humans comprises ZEB1 (or δEF1) and ZEB2 (or SIP1), which are zinc finger TFs (99). ZEB TFs bind at bipartite E-boxes using their zinc-finger domains (100). Both ZEBs actively repress epithelial cell markers, and activate the expression of mesenchymal biomarkers, thus, mediating EMT (101). During physiological conditions, they are primarily expressed in the CNS, heart, skeletal muscle and hematopoietic cells. ZEB1 and ZEB2 can, in part, compensate for each other (102). Nevertheless, in lymphocytes, ZEB1 is mainly found in the thymus during T-lymphocyte development; whereas, ZEB2 is found primarily in the spleen during B-lymphocyte development (102), indicating differences in expression and functionality. The two ZEB TFs can even function antagonistically (103). ZEB2 knockout mice are embryonically lethal (104), indicating that ZEB1 cannot fully compensate for the developmental functions of ZEB2 in its absence.

Multiple signaling molecules control ZEB1 and ZEB2 expression. For instance, estrogen signaling cascades can induce ZEB1 expression (105). Similarly, TGFβ and Wnt/β-catenin signaling are activators of ZEB1 (106). Also, SNAIL1 and TWIST1 cooperatively control ZEB1 expression levels (107). In turn, ZEB1 suppresses multiple genes being involved in the generation and maintenance of epithelial cell polarity, including *CDH1, Lgl2, PATJ*, and *Crumbs3* (108). ZEB1/2 expression in epithelial cells results in EMT and a mesenchymal phenotype, promoting invasion, metastatic dissemination and de-differentiation to a cancer stem cell state (109). The expression of ZEB1 associates with poor clinical outcome in solid tumors (110), including those of the breast (111), colorectum (112) or pancreas (113). ZEB2 expression also associates with poor prognosis and survival in different cancer types (114–116). ZEB1/2 and the miR-200 family expression levels are anti-correlated, with a double-negative feedback loop between them, which is described in the section of microRNAs. Post-translational modifications of both TFs can also modulate their expression levels, an example being the phosphorylation of ZEB1 (117) and the SUMOylation of ZEB2 [reviewed in (118)].

**Figure 2:**
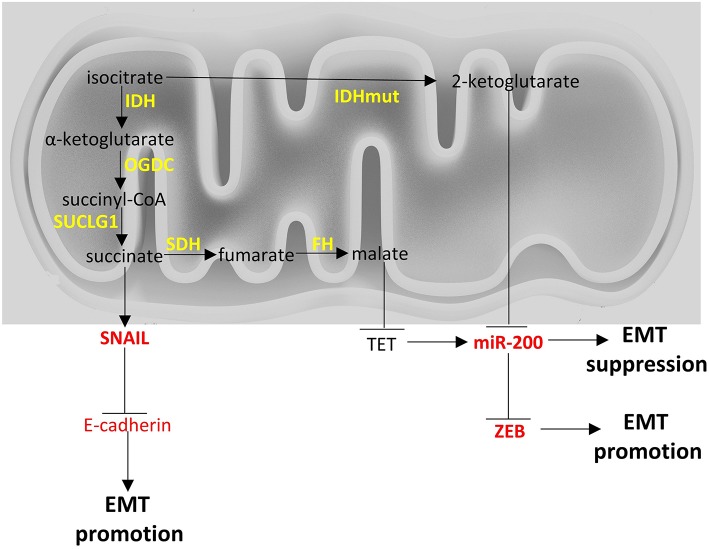
Crosstalk of TCA cycle with EMT factors. Enzymes are depicted in yellow font, EMT-related factors are depicted in red font. → denotes induction; ⊣ denotes inhibition.

**Figure 3:**
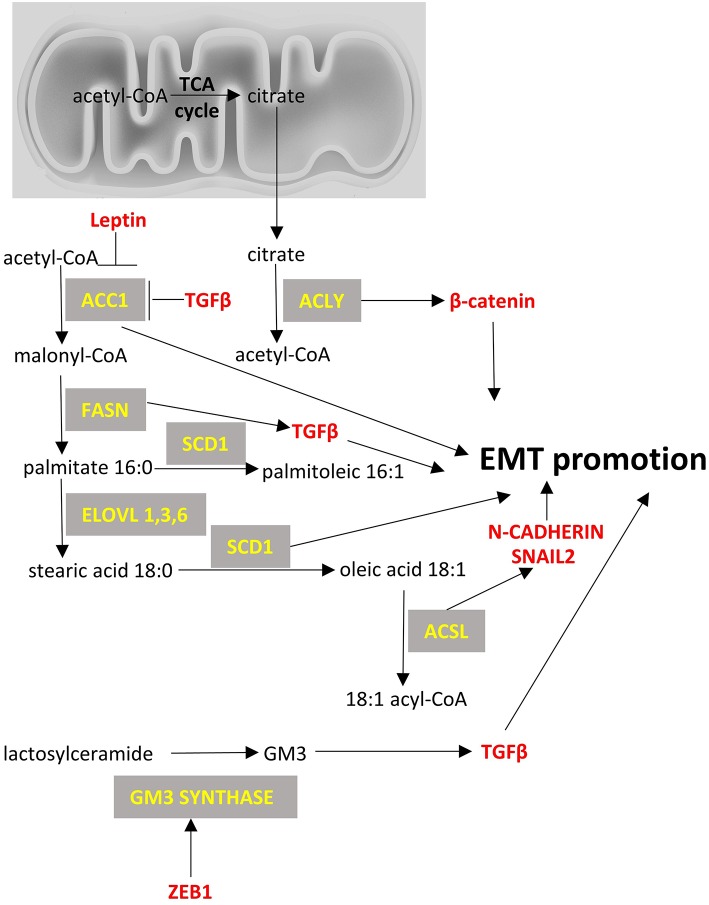
Fatty acid metabolism and EMT factors. Enzymes are depicted in yellow font, EMT-related factors are depicted in red font. → denotes induction; ⊣ denotes inhibition.

From a metabolic point of view, ZEB1 has been recently described to be a central component of adipogenesis (119) in non-cancer cell studies but ZEB1/2 have been more extensively studied in the context of cancer cell metabolism and appear to affect glycolysis (120) (Figure 1), to be affected by TCA cycle byproducts and drive EMT (121) (Figure 2), and to divert glycosphingolipid metabolism (122) (Figure 3).

### Other Non-canonical EMT-TFs

In addition to the thoroughly studied canonical EMT-TFs, a number of other TFs are also implicated in EMT. For instance, Krüppel-like factor 8 (KLF8) promotes EMT in breast (123, 124), ovarian (125) and gastric cancer cell lines (126). In particular, KLF8 resulted in the acquisition of mesenchymal features including enhanced motility, changes in cell morphology and direct inhibition of E-cadherin expression by modulating its promoter (123).

Paired-related homeobox 1 (PRRX1) overexpression activates EMT in certain cancers, including those of the stomach (127), colorectum (128), pancreas (129) or breast (39), and promotes a migratory and invasive phenotype. However, at a later stage of the metastatic process, its expression must be stopped, to promote MET, metastatic colonization and an epithelial phenotype with stem cell features (39). In particular, two isoforms of PRRX1, PRRX1a, and PRRX1b, have distinct functions in EMT and MET in pancreatic ductal adenocarcinoma (36). PRRX1b promotes de-differentiation, invasiveness and EMT; whereas, PRRX1a is involved in differentiation and MET (36).

Forkhead box C2 (FOXC2) is a TF that can induce EMT and thus, indirectly inhibit the expression of E-cadherin (130). Under physiological conditions, FOXC2 plays a role during in embryogenesis, affecting angiogenesis and the development of the muscles, kidney and urinary tract (131), while it also has distinct functionalities in adipocytes (132). Importantly, FOXC2 has roles in the metastatic process through EMT activation in breast, prostate and ovarian cancers (133–136).

Goosecoid (GSC) can indirectly inhibit E-cadherin (130) and is overexpressed in many breast cancers and metastases (137). Another TF, LBX1 (Ladybird homeobox 1) can up-regulate the expression of ZEB1/2 and SNAIL1, promoting cell migration and invasiveness in breast cancer (138). Finally, NF-kB induces EMT by regulating the expression of EMT-TFs, while its inhibition abolishes the metastatic potential of mammary epithelial cells (139).

All these non-canonical TFs that play a role of EMT have not been studied very thoroughly in the very specific content of cancer cell metabolism but some of them like FOXC2 (140) and PRRX1 (141) have established roles in adipocyte metabolism. However, their interaction with ZEB, SNAIL, TGFβ that are major players of EMT means that they can indirectly affect EMT and possibly relevant metabolic processes.

### TFs That Suppress Mesenchymal Toward Epithelial Phenotype

OVOL1 and OVOL2 are zinc-finger TFs involved in the maintenance of the epithelial state (142) and the suppression of the EMT (143). OVOL2 and ZEB1 act as mutual repressors of each other (11). Grainy-head like 2 (GRHL2) is involved in the establishment of epithelial identity (144) and the suppression of EMT (145, 146). It has been shown that GRHL2 suppresses EMT by inhibition of P300, which is required for EMT (146). It also antagonizes TGFβ-induced EMT in gastric cancer (147). Furthermore, knock down of GRHL2 and OVOL2 increases the collective cell migration (148). The pioneer TFs, FOXA1, and FOXA2, are transcriptional activators of epithelial genes, including E-cadherin expression (149). Both FOXA1 and FOXA2 are regulators and antagonists of the EMT and can be down-regulated by SNAIL1, resulting in the inactivation of enhancers at key epithelial genes (150). The TF GATA3 promotes the epithelial phenotype and inhibits metastasis in breast cancer (151). Thus, in addition to the TFs that are EMT inducers there is an opposing set of EMT suppressors. This set of transcription factors that suppress MET are not well described for their metabolic effects with the exception of a study that shows FOXA1 to reduce lipid accumulation in human hepatocytes (152). Their crosstalk though with ZEB1 or SNAIL can be relevant in an indirect way to cancer cell metabolism.

## MicroRNAs

microRNAs (miRNAs) are small (18–24 nt long), non-coding RNAs that can post-transcriptionally fine-tune gene expression by targeting the 3′UTR of mRNA transcripts, leading to their destabilization and degradation. They are initially transcribed by RNA polymerase II into a pri-miRNA and processed by DROSHA to generate the pre-miRNA, which is subsequently exported from the nucleus and processed by DICER to mature miRNA (153). The mature miRNA interacts with the RNA-induced silencing complex (RISC) to target and cleave complementary mRNA molecules. About one third of human genes are recognized and targeted by miRNAs (154), indicating their pervasive regulatory control. Different miRNAs have been found to either promote or inhibit the EMT process through a multitude of functions. To date, over 130 different miRNAs have been implicated in EMT regulation (155) through combinatorial control networks (45). Among miRNA targets, there are multiple EMT-TFs, including SNAIL, TWIST and ZEB1/2 (156). microRNAs have also been described to regulate various metabolic processes including but not not limited to glucose and lipid metabolism in non-cancer cells (157) and have been described to participate in the regulation of metabolic pathways in cancer cells (158).

### The miR-200 Family in EMT Suppression

The miR-200 family members include miR-200a, miR-200b, miR-200c, miR-141, and miR-429, which are clustered in two polycistronic pri-miRNA loci, found in chromosomes 1 and 12, in humans. They can inhibit EMT by targeting the mRNA molecules of EMT promoting factors, resulting in their transcript degradation or translational repression. The miR-200 members share many of their targets, due to the high sequence homology between them in their seed region. Their overexpression leads to an increased E-cadherin expression, the maintenance of the epithelial phenotype and the inhibition of EMT (159). The miR-200 members target ZEB1/2, both of which repress E-cadherin (160–162). Their expression in cell lines results in MET, with the acquisition of epithelial cell morphology, phenotype and biomarkers and the loss of mesenchymal features, including the migratory phenotype and mesenchymal-associated biomarkers (159). ZEB1 can also inhibit the expression of miR-141 and miR-200c, increasing the complexity of this interaction network (160). As a result, ZEB1 and the miR-200 family are components of a mutual inhibition circuit. In cancer, the miR-200 family can suppress metastasis (163). The roles of miR-200 family in the suppression of EMT have been extensively studied in lung cancers (164). The miR-200 family also regulates multiple signaling cascades, including the WNT and Notch pathways (165, 166). *TP53* is the most frequently mutated cancer gene among most cancer types and acts as a tumor suppressor (167). ZEB1 and ZEB2 expression can be downregulated by TP53, which activates miR-200 and miR-192, which in turn repress ZEB1 and ZEB2, resulting in EMT inhibition (168). miR-200 family has been reported to regulate pancreatic β cell survival in type 2 diabetes (169) and to be downregulated in high-fat diet-induced obesity in murine adipose tissue (170). In cancer cells it seems to be directly or indirectly intertwined with glycolysis (Figure 1) and TCA cycle metabolic pathways (Figure 2) (171, 172).

### Other miRNAs in EMT

Other miRNAs can also regulate EMT, apart from the miR-200 family. For example, miR-9 and miR-10b can directly inactivate E-cadherin expression, promoting cell motility and metastasis (173–177). In breast cancer, miR-10b expression in otherwise non-metastatic tumors, promotes metastasis and correlates with clinical outcome (178). Also, silencing of miR-10b inhibits metastasis (179), suggesting its value as a putative therapeutic target. MYC and MYCN activate miR-9 inducing EMT in breast cancer and its expression is correlated with MYCN gene amplification in neuroblastoma (173). In addition, miR-29b and miR-30a inhibit the expression of SNAIL1 (156, 180). In prostate cancer, miR-29b levels are decreased and in prostate cancer cells its expression upregulates epithelial markers and downregulates mesenchymal markers (180). miR-34 and SNAIL1 both negatively control the expression of each other (181). During TGFβ-induced EMT, SNAIL1 suppresses the expression of miR-34. In breast cancer, miR-203 and SNAIL1 also negatively control the expression levels of each other (182). Similarly, there is a double-negative feedback loop between SNAIL2 on one hand, and miR-1 and miR-200b on the other (183). miR-21 has an EMT-promoting activity and is overexpressed in many cancers. It can up-regulate PTEN which in turn phosphorylates EMT factors to inhibit the EMT process (184). miR-23b targets ZEB1 (185). miR-424 is upregulated early during TWIST1- or SNAIL-driven EMT with roles in promoting the mesenchymal transitioning, without altering epithelial attributes (186). miR-205 family downregulates the expression of ZEB1/2 and in conjunction with the miR-200 members, it promotes MET (161). As a result of the above, multiple miRNAs are involved in the regulation of EMT across different cancer types and with a multitude of targets. Even though there is evidence that most of these described miRNAs play some roles in metabolic processes in normal cells, there is no concluding evidence that these, with the exception of miR-200, play specific roles in cancer cell metabolism with relevance to EMT process. Consequently, in the sections on EMT and metabolism below miR-200 is discussed more extensively and is depicted in summary Figures 1, 2.

### Other Long Non-coding RNAs

Long non-coding RNAs (lncRNAs), i.e., non-coding RNAs of >200 nucleotides in length, are also involved in a plethora of biological processes, including EMT (187, 188). Hundreds of lncRNAs are deregulated during EMT (187), either promoting (189–192) or inhibiting it (193–196). Among their functions in EMT control, they can regulate signaling pathways including that of TGFβ (197), they can function as competing endogenous RNAs (ceRNAs) for miRNAs (198) or influence the expression of EMT-associated genes, including EMT master TFs (198–200). A 5′UTR intron at ZEB2 mRNA contains an internal ribosome entry site, which is required for its expression. ZEB2-AS1 lncRNA prevents the splicing of the 5′UTR intron, and enables the production of ZEB2 protein, which then inhibits E-cadherin expression (199). In breast cancer cells, UCA1 lncRNA promotes EMT through the activation of the Wnt/β-catenin signaling pathway (201). Its knock-down induces E-cadherin expression and it also reduces the mesenchymal characteristics of the cells and their invasiveness (201). H19 lncRNA is activated by hypoxia and TGFβ and promotes EMT. In particular, it inhibits E-cadherin expression, increasing the invasiveness of cancer cells and acts as a ceRNA for miR-138 and miR-200a (202, 203). These selected examples demonstrate the plethora and diversity in lncRNA functionalities relevant to EMT. Our understanding of the roles of lncRNAs is rapidly advancing. Expression levels of disparate lncRNAs are being investigated as clinical biomarkers of cancer diagnosis and prognosis (188) and could harbor clinical opportunities for intervention in EMT. Ofcourse, the research on the role of lncRNAs on metabolism is expanding (204) but currently there is not a lot of studies (205) linking them with cancer cell metabolism and specifically the ones that are related with the EMT process.

## Metabolic Pathways Involved in EMT

Metabolic changes during tumor development, of which the most thoroughly described mechanism has been the Warburg effect that facilitates the production of energy mostly from glycolysis and less from oxidative phosphorylation (206), potentiate the aggressive proliferation of cancer cells. However, it is important to note that cancer cells do not exclusively use glycolysis for energy production (207, 208) and studies have shown that oxidative phosphorylation promotion through enhanced mitochondrial biogenesis (209) or function (210) can also promote tumorigenesis progression and EMT. A key work (211) has shown that mesenchymal-like cancer cell lines exhibit a common metabolic gene signature that includes genes related to nucleotide, lipid, amino acid, glycan, carbon, and redox metabolism, and on top of that, known TFs affecting the EMT process, are co-expressed (up- or down-regulated) with these genes. Other individual studies focusing on specific cancers or cell-lines, also point to the same direction. In the following sections we summarize the most important metabolic pathways and their main players that are changing along with EMT, as well as how these can be potentially regulated by known EMT-driving TFs and other regulators. We also discuss if and how these metabolic processes can have an effect on EMT *per se*.

### Glycolysis

Fructose-1,6-biphosphatase 1 (FBP1) is an enzyme that hydrolyzes fructose 1,6-bisphosphate to fructose 6-phosphate and inorganic phosphate and regulates gluconeogenesis. SNAIL1 was found to directly represses the expression of FBP1 in two luminal breast cancer cells lines (212) and this led to enhancement of glycolytic flux, impaired oxygen consumption and reduced reactive oxygen species (ROS) production. FBP1 repression appears to occur due to *de novo* DNA methylation of its promoter. It is also interesting that ectopic overexpression of FBP1 in the SNAIL1-overexpressing cell lines inhibited the initiation of EMT and abrogated the downregulation of E-cadherin that is required for EMT (212). Downregulation of FBP1 has been shown to be a poor prognostic factor in gastric cancer (213) and in aggressive glioblastomas (214), as well indicating the importance of this finding.

Phosphofructokinase 1 (PFK1) is an important glycolytic enzyme that has the opposite function of FBP1; it catalyzes the conversion of fructose 6-phosphate to fructose 1,6-bisphosphate. Its increased expression facilitates the glycolytic flux and it is usually induced under hypoxic conditions as part of a wider transcriptional response induced by hypoxia-inducible factor 1 (HIF-1) (215, 216). Increased HGF signaling has been shown to lead to increased PFK1 activity and to EMT in a human hepatocarcinoma cell line (217). However, in cases where nutrients from cancer cells are depleted, glycolysis is no longer the “preferred” pathway for these cells and the glycolytic flux is diverted to pentose phosphate pathway (PPP) and PFK is repressed. SNAIL, a key EMT-TF has been described to repress PFKP, a major isoform of PFK1 (73). In breast cancer cell lines, under conditions of limited nutrients, it promotes PPP that generates NADPH, a reducing equivalent, and precursors for the synthesis of fatty acids, amino acids and nucleotides (73). In this way, the “stressed” cancer cells can survive in conditions of nutrient deprivation and its metastatic potential increases.

Hexokinases are enzymes that phosphorylate glucose to produce glucose-6-phosphate, which is the first step in most glucose pathways, including glycolysis. Hexokinase 2 (HK2) is the major isoform that is overexpressed in cancers (218) and its depletion can ameliorate the outcomes in a model of hepatocellular carcinoma (219). There are some indications that, under hypoxic conditions, the overexpression of HK2 can facilitate EMT (220). Another hexokinase isoform (HK3) has recently been described to be associated with EMT in colorectal cancers (221). Exact molecular mechanisms have not been described and these data are mainly based on association studies.

Pyruvate Dehydrogenase Kinase 4 (PDK4) is located in the mitochondrial matrix and inhibits the pyruvate dehydrogenase complex via phosphorylation. Thus, it inhibits the conversion of pyruvate to acetyl-CoA decreasing the metabolites flux to tricarboxylic acid cycle, down-regulating aerobic respiration and promoting glycolysis and fat metabolism. PDK4 has been described to have oncogenic roles in human colon cancer cells (222) and its increased levels to be related with aggressiveness and chemoresistance in bladder cancer (223). However, low PDK4 levels were found to be associated with poorer prognosis in a series of non-small cell lung cancer samples (194).

Pyruvate kinase (PK) catalyzes the transfer of a phosphoryl group from phosphoenolpyruvate to ADP, generating ATP and pyruvate, which is actually the last step of glycolysis. A splice variant of PK, PKM2, is expressed in fetal tissues and cancers (224) and has been shown to be part of EMT in human colorectal cancer cells. Specifically, PKM2 translocates in the nucleus during EMT where it represses E-cadherin transcription by interacting with TGFβ-induced factor homeobox 2 (TGIF2) (225). This role of PKM2 can be described as non-canonical, as it does not refer directly to the classic role of this enzyme (catalysis of glycolysis) but it also shows that metabolism-related enzymes can acquire alternative functions in cancer cells that may be critical in the fate of cancer cells. Of course there are several instances where it is shown that PKM2 expression is enhanced in cancers and favors the glycolytic pathway and potentially the metastatic potential such as in pancreatic ductal adenocarcinoma tissues and cell lines (226).

Enolase 1 (ENO1) catalyzes the conversion of 2-phosphoglycerate to phosphoenolpyruvate and it is usually overexpressed in cancers, such as those in head and neck or lung (227, 228). Lung adenocarcinomas show increased ENO1 expression and its silencing represses EMT in the relevant cell lines models (229). Proteomic analysis in gastric cancer cells has revealed that ENO1 is central to a protein-protein interaction network that regulates tumor growth and metastasis (230).

Phosphoglucose isomerase (PGI) converts glucose-6-phosphate to fructose-6-phosphate. Interestingly this protein can also be secreted by cancer cells and act as a cytokine (autocrine motility factor; AMF) promoting migration, invasion and metastasis (231). PGI has also been shown to promote EMT in breast cancer cells by repressing miR-200 and inducing ZEB1/2 (171) and silencing of PGI expression promotes mesenchymal to epithelial transition in human lung fibrosarcoma cells (232).

Aldolase A (ALDOA) catalyzes the conversion of fructose-1,6-bisphosphate to glyceraldehyde 3-phosphate and dihydroxyacetone phosphate. ALDOA is usually overexpressed in cancers and it is usually associated with poor prognosis (233). Downregulation of ALDOA in squamous lung carcinoma lines led to reduced expression of mesenchymal markers (234). Its overexpression in colon cancers is also associated with worse outcomes and also leads to EMT as shown by RNA-seq based transcriptomics analysis (235). Supportive of this role of ALDOA are other studies showing that silencing of ALDOA increased E-cadherin (epithelial marker) and decreased N-cadherin (mesenchymal marker) in pancreatic cancer (236) and bladder cancer cell lines (237).

Glucose transporters 1 and 3 (GLUT1, GLUT3) facilitate the entrance of glucose in cells in an insulin-independent manner. GLUT1 is expressed at different levels in all tissues and mostly in fetal tissues while GLUT3 is most abundant in neurons. Cancer cells usually overexpress GLUT1 and GLUT3 to facilitate the uptake of glucose independent of insulin levels, and high levels of GLUT1 and GLUT3 are usually associated with poor prognosis (238, 239). In laryngeal cancer cells GLUT1 expression correlated with Vimentin and N-cadherin expression that are markers of EMT (240). GLUT3 has been found overexpressed in mesenchymal cells of non-small cell lung cancer and ZEB1 can induce GLUT3 expression in these cancer cells (241), indicating that GLUT3 is an important component of EMT. In Figure 1 we briefly summarize the role of the glycolytic pathway in the EMT process.

### The Tricarboxylic Acid Cycle (TCA) Cycle

Fumarate hydratase (FH) converts fumarate to malate. Loss of functions mutations of *FH* lead to leiomyomatosis, renal cancer and pheochromocytomas (172, 242). Accumulation of fumarate, due to these mutations, can lead to EMT in renal cancer cells. Specifically, fumarate can inhibit Ten-Eleven translocation (TET)-mediated demethylation of the regulatory region of miR-200. Hence, fumarate can ultimately inhibit miR-200 family expression and thus, abrogate miR-200-mediated EMT suppression (172).

Succinate dehydrogenase (SDH) catalyzes the oxidation of succinate to fumarate. Loss of function mutations of *SDH* are found in paragangliomas, gastric stroma tumors and pulmonary chondromas (243, 244). Metastatic pheochromocytomas and paragangliomas with reduced SDH expression due to *SDHB* mutations, show an EMT signature based on transcriptomics analysis and increased SNAIL 1/2 protein expression (245). Interestingly, breast cancer cell lines undergoing EMT show reduced SDH expression and hepatocellular carcinoma cell lines with reduced SDH expression show increased expression of EMT markers (246), indicating there may be a link between EMT and SDH (247) with molecular mechanisms that warrant further investigation. There is a hint that accumulation of succinate due to *SDH* mutations can induce EMT with a similar mechanism with fumarate (172).

Isocitrate dehydrogenases (IDH) catalyze the conversion of isocitrate to α-ketoglutarate. Three isoforms exist in humans: IDH1 and IDH2 which are NADP^+^ dependent, and are unrelated to IDH3. IDH1 and IDH2 catalyze reversible reactions while the reaction catalyzed by IDH3 is not reversible and is subject to allosteric modifiers (248). Mutations of *IDH1* and *IDH2* have been described in cancers and specifically in gliomas (249) and leukemia (250). *IDH1* and *IDH2* mutations render the enzymes to mainly produce 2-hydroxyglutarate, instead of α-ketoglutarate. Accumulation of 2-hydroxyglutarate leads to an EMT phenotype that is dependent on upregulation of ZEB1 and downregulation of miR-200 family (121). In colorectal cancer cell lines 2-hydroxyglutarate increased ZEB-1 expression by trimethylation of histone H3 lysine 4 of the promoter region of ZEB1 (251). In Figure 2 a brief visual summary of the main TCA cycle interactions with EMT is provided.

### Lipid Metabolism

*De novo* lipogenesis is the synthesis of fatty acids from non-lipid precursors (mostly carbohydrates in the form of acetyl-CoA). Ultimately, the fatty acids are esterified to glycerol to form triglycerides. Cancer cells usually show increased lipogenesis (252) and this is the reason why lipogenesis has been proposed as a target for cancer treatment. Little is known though for the role of lipogenesis genes in EMT. We summarize below the current knowledge regarding them.

Acetyl-CoA carboxylase (ACC) catalyzes the carboxylation of acetyl-CoA to malonyl-CoA. Two ACC isoforms exist: ACC1, that is found in cytoplasm and regulates *de novo* lipogenesis; and ACC2, that is found at the mitochondrion membrane and mainly regulates fatty acid oxidation. ACC1 has been found to be overexpressed in cancers, such as those of the breast (253) and liver (254), and blocking of ACC1 has been shown to reduce lung tumor growth in mice (255). However, there is limited data on the role of ACC1 in EMT, with the exception of a relatively recent paper on breast cancer cells, that suggested an alternative non-canonical role for ACC1 in EMT. Specifically, it was shown that leptin and TGFβ can inhibit the activity of ACC1 through AMPK-phosphorylation of ACC1 at Ser79 and promote EMT (256). It was suggested that this effect should be mediated by accumulation of acetyl-CoA because of ACC1 inhibition and by the concomitant increased acetylation of SMAD2 that mediates the TGFβ-induced EMT. It, thus, seems that even though ACC1 expression is found increased in some cancers including breast cancer, it does not necessarily mean that it would be a good treatment approach to silence ACC1 as it can increase metastatic potential by favoring EMT.

Fatty acid synthase (FASN) is a multifunctional protein with its main function being the synthesis of palmitate from acetyl-CoA and malonyl-CoA. In some cancers, a fusion of *FASN* and Estrogen receptor α (*ER-a)* genes has been described that may play a role in estrogen signaling (257). Overexpression of FASN has been described in cancers like gastrointestinal stromal tumors (257), breast (258), ovarian (259), and lung cancers (260). It has been proposed that enhanced FASN expression in cisplatin-resistant non-small cell lung cancer cells promotes EMT through TGFβ signaling (260). Other smaller studies have suggested that FASN may mediate EMT, but they have not provided any mechanistic insight (261).

Stearoyl-CoA desaturase-1 (SCD-1) is an enzyme anchored in endoplasmic reticulum that catalyzes the formation of monounsaturated fatty acids (oleate and palmitoleate from stearoyl-CoA and palmitoyl-CoA, respectively). SCD-1 is overexpressed in cancers such as lung adenocarcinoma and its increased expression correlates with poor prognosis (262). Silencing of SCD-1 in breast cancer cells has led to impairment of their EMT-like behavior and to decreased nuclear localization of β-catenin, a known EMT mediator (263). ATP citrate lyase (ACLY) converts mitochondrially-derived citrate into acetyl-CoA and oxaloacetate providing the acetyl-CoA necessary for lipogenesis. ACLY is usually overexpressed in cancers (264, 265) and has been shown to promote EMT phenotypes in colon cancer cells at least partly through β-catenin signaling (266). Inhibition of ACLY has been proven effective to prevent EMT induced by ambient fine particulate matter (PM2.5) (267) and to reverse EMT phenotype in a lung adenocarcinoma cell line (268).

Acyl-CoA Synthetase Long Chain Family Member (ACSL) catalyzes the formation of fatty acyl-CoA from fatty acids and isoforms 1, 3, and 4 are more often overexpressed in cancer cells and specifically in colorectal (269), breast (270) and prostate cancers (271). Each isoform uses different substrates e.g., ACSL1 uses oleate and linoleate, ACSL3 uses myristate, palmitate, arachidonate and eicosapentaenoate and ACSL4 arachidonate (272). This activation of fatty acids by ACSL is a necessary step for the synthesis of cellular lipids as well as the β-oxidation. In colon cancer cells increased expression of ACSL1 and ACSL4 is associated with EMT features of these cells (273). The mechanism is not clear but one report suggests that this offers a metabolic advantage in the cancer cells by making them more energy efficient and by increasing the expression of SNAIL2 and N-cadherin (274).

Last, the metabolism of complex lipids and specifically glycosphingolipids has been shown to affect EMT. GM3 synthase converts lactosyl ceramide to a simple ganglioside called GM3 which is known to promote EMT by interacting with TGFβ receptors (275). It has been shown that ZEB1 can induce GM3 synthase gene by binding to its promoter and by repressing the micro-RNA mediated suppression of GM3 synthase in human lung cancer cells (122). Further work is needed to evaluate the importance of this regulation of glycosphingolipids metabolism in EMT in various types of cancer. Figure 3 summarizes the main lipid metabolism pathways that interact with EMT process.

### Amino Acid Metabolism

Amino acid metabolism is essential for the maintenance of cellular homeostasis. In cancer cells there is an increased need for nitrogen for biosynthetic reactions, amino acids are consumed quickly and there is increased demand for non-essential amino acids that exceeds the supply (276). It is also impressive that in most cancer cells glutamine is the second highest nutrient in demand after glucose (277). Herein, we focus on the amino acids of glutamine, asparagine and cystine that have been described to be at least partly implicated in EMT in cancers.

Glutamine is the most abundant amino acid in serum and is highly consumed by many cancer cells. It provides also the major source of α-ketoglutarate in glutamine-dependent cancer cells to be used in TCA cycle through a process called glutaminolysis (278). Glutaminases 1 and 2 (cytosolic GLS1, mitochondrial GLS2) catalyze the hydrolysis of glutamine to glutamate and ammonia. GLS1 can be induced by TGFβ and Wnt and can promote EMT in a SNAIL-dependent manner while silencing of GLS1 prevents EMT (279). In contrast with GLS1 which is ubiquitously expressed, GLS2 is mainly expressed in brain, liver and pancreas and is inversely associated with EMT in breast cancer and hepatocellular carcinoma cells (279, 280). Interestingly, GLS2 levels are inversely correlated with GLS1 levels in breast cancer and it seems that at least in breast cancer cells GLS2 downregulation is the result and not the driver of EMT; silencing of FOXC2 led to increased levels of GLS2, did not affect GLS1 levels and led to inhibition of EMT (280). These observations mean that there may be required a fine tuning of glutaminolysis in different compartments of the cancer cells, reflected by the GLS1/GLS2 ratio, to support EMT and interfering with this can be a promising method for EMT inhibition.

Asparagine is a non-essential amino acid in humans and its abundance has been associated with EMT and the metastatic potential of breast cancer cells. Increased intake of asparagine with diet or increased asparagine synthetase activity led to increased incidence of metastases whereas reduced diet intake of asparagine or decreased asparagine synthetase activity or treatment with L-asparaginase reduced metastatic potential without affecting the growth of the primary tumor (281). Asparagine can become an essential amino acid in cases of glutamine deprivation in the tumor microenvironment so as to maintain protein synthesis and cell proliferation (282). Proteins that are upregulated during EMT have a ~20% higher asparagine content (281). Thus, it is rational to hypothesize that reduced asparagine availability would inhibit EMT at least at the translational level. However, it not clear how asparagine can transcriptionally regulate EMT genes like TWIST or N-cadherin (94). Further investigation is warranted to unravel all the potential mechanisms of asparagine's contribution to EMT.

**Figure 4:**
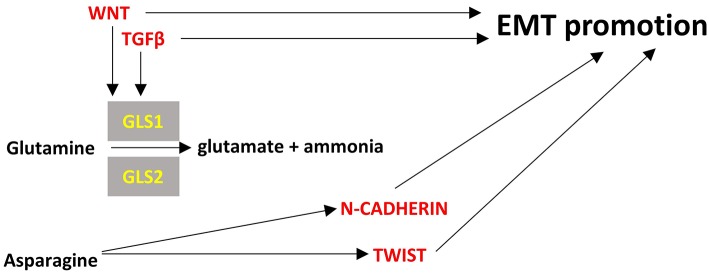
Points of convergence between amino acid metabolism and EMT. Enzymes are depicted in yellow font, EMT-related factors are depicted in red font. → denotes induction.

Cystines formed by the oxidation of two cysteine molecules and their link by a disulfide bond. It is the main circulating form of cysteine that can be uptaken by cells. Cancer cells can become “addicted” to cysteine (283) and their reliance on cystine may be associated with EMT (284). Overexpression of miR-200c, that inhibits EMT, in cystine-addicted breast cancer cells resulted in these cells being less vulnerable to cystine deprivation (284). This is an indication that cystine can become an essential amino-acid during EMT at least in breast cancer cells but it is not known if and how it can affect EMT. Figure 4 summarizes briefly the interplay of amino-acid metabolism with EMT process.

## Existing or Promising Methods for Intervening in the EMT Process

Cancer is highly prevalent and remains a leading cause of death. The identification of novel treatments for the primary tumor as well as the discovery of potent inhibitors of metastasis is imperative. EMT and partial EMT can confer metastatic and stem cell properties to tumor cells (13, 285) and are correlated with the clinical outcome for cancer patients across multiple cancer types. It has also been demonstrated that EMT is linked to drug treatment resistance for multiple drugs (286, 287) and to multidrug resistance phenotype (288). EMT could be an attractive target to halt invasive and potentially metastatic cancer cells and to address treatment resistance. As a result, research in the development of EMT inhibitors for combinatorial cancer treatments is pivotal. In this section, we discuss multiple promising EMT targets including targets that link metabolic pathways with EMT.

The activity of EMT-TFs can be modulated by a number of kinases, which are therefore putative targets for intervention. In pancreatic cells, SB431542 blocks TGFβ-induced EMT by targeting the activity of TGFβ receptor kinase (289). In breast cancer cells, AG1478 targets EGFβ receptor kinase to halt EMT induction (290). Another small molecule inhibitor, BGB324 (also known as R428), blocks Axl kinase and inhibits metastasis (291). The signaling pathways involved in EMT are also putative therapeutic targets. TGFβ has been a target in several cancer types with Fresolimumab (GC-1008), which is a monoclonal antibody, in trials targeting EMT (292–294). Notch-2 is a signaling factor that promotes EMT. In pancreatic cancer, inactivation of Notch-2 by γ-secretase inhibitor IX resulted in selective inhibition of EMT (295). The mesenchymal phenotype of cancer cells has been the target of multiple additional intervention strategies. Withaferin A promotes the degradation of Vimentin (296) and can halt the migratory and invasive properties of cancer cells, therefore inhibiting the metastatic process. Antibody development against mesenchymal factors is also being pursued. For instance, an antibody raised against N-cadherin inhibits prostate cancer growth and metastasis (297). Certain miRNAs can halt the EMT induction while others promote it. In pancreatic cancer, miR-200 and let-7 upregulation by the natural compounds 3,3'-diindolylmethane and isoflavone results in a partial reversal of the EMT phenotype (298). Targeted inhibition of miR-21 has also been pursued with the development of a small molecule inhibitor, AC1MMYR2, which reverses the EMT process (299). Also, recent studies are unraveling the contribution of different lncRNAs in EMT regulation and have indicated that many lncRNAs could be utilized as clinical biomarkers such as prognostic and diagnostic biomarkers of metastasis and as potential therapeutic targets to inhibit cancer metastasis (188, 300).

Targeting metabolic pathways important for EMT has also been considered an alternative means of halting the EMT process (301). However, as most of these metabolic pathways are also essential for the survival of non-cancer cells, it is important to target as specifically as possible a certain pathway and ideally focus the treatment on the tissue and cells of interest and of course on the specific type of cancer. The metabolic pathways we describe herein in the context of EMT (glycolysis, TCA cycle, lipid metabolism, amino acid metabolism) have also been described as targets for treatment of cancer (302) and thus we could make use of the knowledge on targeting these pathways and affect the metabolism-dependent EMT. Specifically, glycolysis can be targeted at various levels (303, 304). For example, small molecule inhibitors of GLUT1 like fasentin (305), HK2 inhibitors like 3-bromopyruvate, and lonidamine (306) and PKM2 inhibitors (307) could be good candidate substances to be tested for EMT inhibition purposes. TCA cycle enzymes like IDH have been targeted in leukemias with inhibitors (e.g., AGI-6780) (308) and could also be the focus of studies on EMT. Lipogenesis inhibitors such as specific ACC1 inhibitors (309), SCD1 inhibitors (310) and ACLY inhibitors (311). Furthermore, methods to interfere with amino acid metabolism and specifically glutamine, asparagine and cystine would require even more fine tuning and targeted approaches given the differential role these amino acids may play in the original cancer and in the existing metastasis (94).

Last, the ideal scenario of intervening in the connection between EMT and cancer and especially in the possible hybrid mesenchymal-epithelial states of cancer cells warrants further investigations. Such research studies have shown for example that it is more effective to suppress TGFβ-driven EMT through targeting elements of the feedback loop between SMAD mediators of TGFβ signaling and EMT components in parallel and this intervention also inhibits these highly metastatic “hybrid” cancer cells (312). A combination of bioinformatics analyses (313) and single-cell sequencing studies (314) along with clinical cohorts of specific cancers (208) will be instrumental to unravel critical targets of EMT pathways and metabolism in parallel for the maximal effect on inhibiting EMT, haltering cancer progression and avoiding the formation of hybrid-state cancer cells.

## Concluding Remarks

In this review, we summarize the current knowledge on: (a) main molecular players underlying the EMT process; specifically transcription factors SNAIL, TWIST, ZEB, other non-canonical transcription factors and non-coding RNAs, and (b) the major metabolic pathways associated with EMT (glycolysis, TCA cycle, lipid and amino acid metabolism). We also review how these pathways can crosstalk with the molecular players. It is gradually becoming evident that there is a network of factors being affected by (and affecting) the metabolism of the pre-cancer or cancer cells, which change the fate of the cells through EMT, with major implications in cancer development. In Figures 1–4 we summarize the points where factors that affect EMT interplay with metabolic pathways. It is important to note that the data we summarize here emanate from studies on various cancer cell types that are noted in each instance. Thus, although cancers share certain common pathways, researchers should be cautious not to extrapolate results from one cancer type to another, but perform similar studies to their cancer of interest, before arriving to a conclusion. The metabolic features of cancer cells are an expanding field of study, as they are distinct from non-cancerous cells and could harbor therapeutic opportunities for intervention in EMT. Of course, suppressing EMT is an emerging prospect of preventing metastases, but it needs to be taken into consideration that this process represents a dynamic spectrum and once cancerous cells invade a tissue, they can undergo MET (315). Therefore, interference with the EMT process could promote metastasis if not targeted specifically at the site of origin.

## Author Contributions

IG-S, DC, and AZ conceived the study and wrote the review. VK contributed to writing and discussion. AZ supervised this work and paid the publication fees.

### Conflict of Interest

The authors declare that the research was conducted in the absence of any commercial or financial relationships that could be construed as a potential conflict of interest.
